# Latest Advances of Virology Research Using CRISPR/Cas9-Based Gene-Editing Technology and Its Application to Vaccine Development

**DOI:** 10.3390/v13050779

**Published:** 2021-04-28

**Authors:** Man Teng, Yongxiu Yao, Venugopal Nair, Jun Luo

**Affiliations:** 1 Key Laboratory of Animal Immunology, Ministry of Agriculture and Rural Affairs & Henan Provincial Key Laboratory of Animal Immunology, Henan Academy of Agricultural Sciences, Zhengzhou 450002, China; tm135@aliyun.com; 2 UK-China Centre of Excellence for Research on Avian Diseases, Henan Academy of Agricultural Sciences, Zhengzhou 450002, China; 3 The Pirbright Institute & UK-China Centre of Excellence for Research on Avian Diseases, Pirbright, Ash Road, Guildford, Surrey GU24 0NF, UK; yongxiu.yao@pirbright.ac.uk (Y.Y.); venugopal.nair@pirbright.ac.uk (V.N.); 4 College of Animal Science and Technology, Henan University of Science and Technology, Luoyang 471003, China

**Keywords:** gene editing, CRISPR/Cas9, virus, gene function, vaccine

## Abstract

In recent years, the CRISPR/Cas9-based gene-editing techniques have been well developed and applied widely in several aspects of research in the biological sciences, in many species, including humans, animals, plants, and even in viruses. Modification of the viral genome is crucial for revealing gene function, virus pathogenesis, gene therapy, genetic engineering, and vaccine development. Herein, we have provided a brief review of the different technologies for the modification of the viral genomes. Particularly, we have focused on the recently developed CRISPR/Cas9-based gene-editing system, detailing its origin, functional principles, and touching on its latest achievements in virology research and applications in vaccine development, especially in large DNA viruses of humans and animals. Future prospects of CRISPR/Cas9-based gene-editing technology in virology research, including the potential shortcomings, are also discussed.

## 1. Introduction

The engineering of viral genomes is important in many aspects of virus research, including studies of the structure and function of viral protein-coding genes and non-coding RNAs, virus–host interactions, gene therapy applications, and the development of recombinant vaccines. In addition to the classical recombination and selection of recombinant viruses in virus-infected eukaryotic cells used previously [1], three main kinds of technology systems have been developed for viral genome engineering: (a) the bacteria-based homologous recombination system (BHRs) [2,3], (b) the bacterial artificial chromosome system (BACs) [4], and (c) the hybrid yeast–bacteria cloning system (HYCs) [5]. The BHR system can be divided into three types: homologous recombination, site-specific recombination, and transposition recombination, which have played an important role in the study of bacterial genetic variation. However, this has not proved to be very efficient for the operation and reorganization of viral genomes. BAC systems are widely used in virus research and their editing efficiency is relatively high [6,7,8,9,10,11,12,13]. Viral genomes need to be first cloned into BAC plasmids in the early stage. The technique of BAC mutagenesis is relatively easy for viruses with small genomes (<10 kb), such as the human immunodeficiency virus (HIV) and hepatitis C virus (HCV). However, for viruses with large genomes (>100 kb), such as herpesviruses and baculoviruses, the construction of BAC clones with full-length genomes is time consuming and laborious. Further, the insertion of drug selection marker genes or BAC plasmid fragments into the viral genome may alter the biological characteristics of the recombinant virus. Usually, it is necessary to use recombination mutagenesis to remove the artificial genetic elements from the recombinant viral genomes. The HYC system is often used to construct various viral vectors, such as adenoviral (AdV) [5] and lentiviral (LeV) vectors [14]. Each of these three systems has its own advantages and/or disadvantages and can use distinct strategies to realize the reconstitution of the viral genome, but to some extent, they are all time consuming, laborious, and/or inefficient. Recently, the new generation of gene-editing technology based on the clustered regularly interspaced short palindromic repeat (CRISPR) and CRISPR-associated (Cas) systems have been well developed and widely applied in many aspects of biological research in humans, animals, plants, and even in viruses. CRISPR/Cas has been demonstrated as a more powerful, simple, efficient, and straightforward approach for editing viral genomes with immense research potential. Herein, we have briefly reviewed the origination, functional principle of the CRISPR/Cas system and focused on the latest advances of CRISPR/Cas9 system-based gene editing in virology and its applications in antiviral vaccine development, especially in large DNA viruses of animals. In addition, future prospects of this innovative technique, including the potential shortcomings in virology research, are also discussed.

## 2. Origin of CRISPR/Cas System

The CRISPR/Cas system is an important part of adaptive immune mechanisms of bacteria and archaea, which is used to resist foreign genetic plasmids and phage invasion. In 1987, the structure of CRISPR was first reported [15], and then similar structures were identified in different bacteria and archaea species. The CRISPR/Cas system was proposed to be abbreviated as ‘CRISPR’ in 2002 [16]. In 2005, CRISPR structures and Cas proteins were speculated to have immune defense function and possibly play an important role in protecting genetic factors [17]. Subsequently, the CRISPR/Cas system is proposed to provide resistance against viruses in prokaryotes, which specifically recognizes and binds the DNA of phage through CRISPR RNA (crRNA) and guides Cas proteins to recognize and cleave exogenous DNA through the trans-activating of crRNA (tracrRNA) [18]. According to different Cas protein families and the principles of effector module design, CRISPR/Cas systems have been divided into two classes, with multi-subunit effector complexes in Class 1 and single-protein effector modules in Class 2 [19]. The CRISPR/Cas9 system, belonging to the Class 2 CRISPR/Cas system, is mainly composed of crRNA and Cas9 protein, and only needs a single guide RNA (sgRNA) to precisely cleave the target genes [20,21]. In 2012, the Cas9-crRNA complex was proved to have the ability to cut target DNA in vitro, and the double-stranded breaks (DSBs) occurred at three nucleotides upstream of protospacer adjacent motif (PAM) sequence, realizing it as the first gene-editing tool in a test tube [22]. Subsequently, Charpentier and Doudna’s groups reported the combination of crRNA and tracrRNA into a single sgRNA, which can more efficiently help Cas9 to play its editing role in vitro [23]. In 2013, Zhang’s lab first applied the CRISPR/Cas9 system to perform genome editing in eukaryotic cells [24]. From then on, the new generation of CRISPR gene-editing technology, especially represented by the CRISPR/Cas9 system, has been well developed and widely applied in the field of life sciences, such as to produce gene-edited animal models, gene therapy to treat genetic disease, and animal and plant genetic trait improvement and biological breeding [25,26,27,28,29,30,31]. In 2020, for the epoch-making technological innovation and great contribution to life sciences, two scientists, Charpentier and Doudna, devoted most to the CRISPR/Cas9-based gene editing were awarded the Nobel prize in Chemistry.

## 3. Principle of CRISPR/Cas System

The CRISPR/Cas9 system can target any kind of gene or genomic region, allowing site-directed gene editing such as mutation, knockout, insertion, and deletion (indel). The functional complex consists of the Cas9 nuclease and a sgRNA, which is composed of a crRNA and a tracrRNA. The chimeric sgRNA directs the Cas9 nuclease to bind to the target DNA fragment followed by 5′-NGG-3′ (PAM) [24], and then stimulates double-stranded DNA cleavage activity of the Cas9 nuclease to produce a DSB. The DSB is mainly repaired by non-homologous end joining (NHEJ) DNA replication or by the homology-directed repair (HDR) pathway. A frameshift mutation in the protein-coding region may affect the normal transcription and translation of the target gene, resulting in the loss of protein function. If the foreign sequence flanked by homologous arms of genomic sequences on both sides of the cleavage site is introduced into the cells when the Cas9 nuclease cuts the double-stranded DNA, the foreign gene will be inserted into the specific sites of the cell genome by HDR pathway, and thus repairing the indel of the target gene [23]. Because of the controllable and flexible selection of guide RNA (gRNA) target sites, the CRISPR/Cas9 system has been developed rapidly and applied widely in the genome editing of mammalian cells, vertebrates, plants, and even viruses.

## 4. CRISPR/Cas System in Virology Research

**Figure 1:**
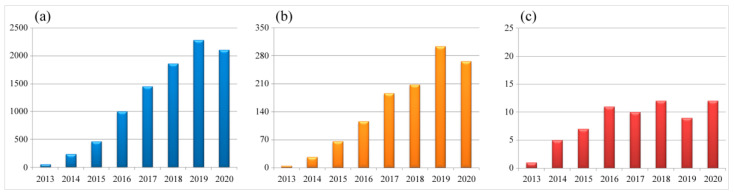
Numbers of scientific reports focused on CRISPR and gene editing during 2013–2020. (**a**) Total number of publications in the field of life sciences. (**b**) Total number of publications in the field of virology. (**c**) Numbers of virus species being studied each year. Data are collected from the NCBI PubMed database (27th March 2021), searched with the keywords CRISPR plus gene editing or CRISPR plus virus and gene editing, respectively.

**Figure 2:**
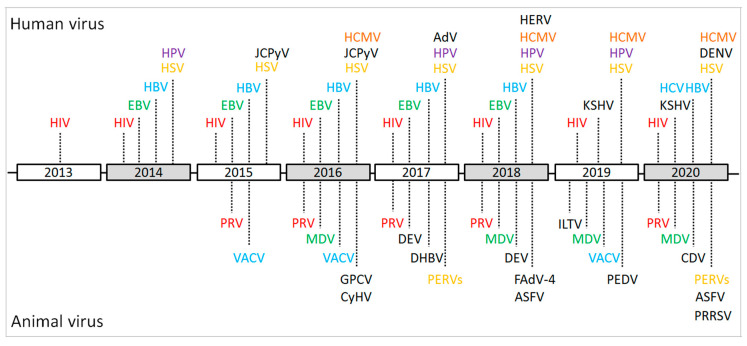
Schematic of the important events and achievements of the CRISPR/Cas9-based gene editing of viral genomes during 2013–2020. HIV: human immunodeficiency virus; EBV: Epstein–Barr virus; HSV: herpes simplex virus; HBV: hepatitis B virus; HCV: hepatitis C virus; HPV: human papillomavirus; HCMV: human cytomegalovirus; JCPyV: JC polyomavirus; KSHV: Kaposi’s sarcoma-associated herpesvirus; AdV: Adenovirus; HERV: human endogenous retrovirus; DENV: dengue virus; PRV: pseudorabies virus; VACV: vaccinia virus; MDV: Marek’s disease virus; GPCV: guinea pig cytomegalovirus; CyHV: cyprinid herpesvirus; DEV: duck enteritis virus; DHBV: duck hepatitis B virus; PERVs: porcine endogenous retroviruses; ASFV: African swine fever virus; FAdV-4: Fowl Adenovirus 4; ILTV: infectious laryngotracheitis virus; PEDV: porcine epidemic diarrhea virus; CDV: canine distemper virus; PRRSV: porcine reproductive and respiratory syndrome virus.

In the last decade, the number of publications describing CRISPR and gene-editing-related research in both life sciences and virology has dramatically increased (Figure 1). In particular, the CRISPR/Cas9 system is not only widely used for the gene editing of organisms with a cellular structure, but also applied to some non-cellular organisms, such as DNA virus and some fragments of RNA virus that are integrated into the host genome. As briefly demonstrated in Figure 2, the CRISPR/Cas9 system has been successfully applied to the gene editing of many different virus species, mainly focusing on antiviral therapy, functional study of viral virulence factors, and the reconstitution of commonly used viral vectors for genetically engineered vaccine development. In addition, the CRISPR/Cas9 system has also been used to study the virus–host interactions through whole-genome screening and identifying the host factors that are essential for virus replication.

### 4.1. Functional Study and Gene Therapy of Virus Using CRISPR/Cas9-Based Gene-Editing Technology

#### 4.1.1. HIV Gene Editing Using CRISPR/Cas9 System

As the CRISPR/Cas9 system can achieve efficient gene editing in biological organisms with cellular structure, its use for editing viral genes integrated into the host genome is attractive as a research tool. It is well known that genomes of HIV and other retroviruses can permanently integrate into the host genome, from where they can be activated to produce an infectious virus that can pose new threats to the host. In 2013, a specific gRNA and CRISPR/Cas9 nuclease complex was designed and transfected into Jurkat cells to target the integrated HIV-1 long terminal repeat (LTR) [32]. The complex efficiently cleaved and mutated the LTR target sites. More importantly, the internal viral genes can also be removed from host chromosomes, indicating that the CRISPR/Cas9 system can be used as a potential tool for curing HIV-1 infection. This was the first report of the CRISPR/cas9 system being successfully applied to viral gene editing. Subsequently, the conserved site of the HIV-1 LTR U3 promoter region was chosen as a new gRNA target for CRISPR/Cas9-based therapeutic gene editing of integrated HIV genomes in microglial, promonocytic, and T cells, where the Cas9 nuclease was successful in the complete excision of the 9709-bp fragment of integrated proviral DNA that spanned from its 5′ to 3′ LTRs [33]. In recent years, there has been significant progress in HIV-1 research utilizing the CRISPR/Cas9 system. For example, the CRISPR/Cas9 system applied to edit multiple targets of the HIV-1 genome was shown to improve the efficiency of knocking out and destroying the non-integrated precursor viral genome [30]. It was also demonstrated that the use of two effective gRNA combinations targeting different regions of the HIV genome can prevent virus replication and escape [34]. The lentivirus-expressed *Staphylococcus aureus* Cas9 (saCas9)/gRNAs composed of multiple gRNAs targeting the conserved region in LTR and viral domain of HIV-1 effectively removed the latent HIV-1 virus, inhibited virus reactivation, and significantly improved the efficiency of destroying the HIV-1 genome [35]. More recent work and findings on gene therapy and editing of HIV-1 have been thoroughly reviewed and reported elsewhere [36,37,38,39,40]. In conclusion, these studies have demonstrated that the CRISPR/Cas9 system can be successfully applied to target and edit the HIV-1 genome, to inhibit HIV-1 infection, eliminate the virus, and even to induce the transcriptional activation of the latent virus to eliminate the virus, showing its potential use for HIV-1 therapy.

#### 4.1.2. Oncogenic Virus Gene Editing Using CRISPR/Cas9 System

For human beings, about one-fifth of tumors are caused by a virus infection, of which the oncogenic viral genes directly promote the occurrence and development of tumors [41]. The hepatitis B virus (HBV) can cause acute and chronic hepatitis B, possibly leading to liver cancer. Researchers first tried to specifically cut the covalently closed circular DNA (cccDNA) of HBV in infected nuclei using the CRISPR/Cas9 system and recommended it as a potential choice for clinical therapy [42]. In addition, some scholars have designed eight gRNAs to target HBV of genotype A and significantly reduced the production of HBV core and surface proteins (HBcAg and HBsAg) in Huh-7 cells [43]. Among these gRNAs, two were identified to be effective. Subsequently, single or combined gRNAs targeting the regulatory region of HBV of genotypes A–D were investigated, and all the gRNAs could significantly reduce the production of HBsAg or HBV e antigen (HBeAg) in the culture supernatant. The efficacy of dual gRNAs in suppressing the production of the two antigens (Ags) increased significantly compared to single gRNA (>80%). Interestingly, the dual gRNAs (gRNA-5/gRNA-12) combination efficiently inhibited the expressed template of HBV and destroyed cccDNA reservoirs in HepAD38 cells [44]. Simultaneously, the CRISPR/Cas9 system was also applied to target HBsAg or HBV X protein (HBx) in cell culture and in animal experiments, respectively, and demonstrated that the expression levels of HBsAg in cell culture supernatant and mouse serum were both decreased [45]. Recently, a large number of studies and similar data have been reported on using the CRISPR/Cas9 system to target HBV genes [46,47,48,49,50,51,52,53,54,55,56,57], implying that CRISPR/Cas9-based gene editing may be a potential therapeutic method for HBV infection.

High-risk human papillomavirus (HR-HPV) is recognized as the main cause of cervical cancer. HPV encodes the oncogenic genes E6 and E7, which play important roles in maintaining the malignant phenotype of cervical cancer cells. In 2014, specific CRISPR/Cas9-gRNA complexes targeting the HPV-16 E7 gene were first reported in HPV positive SiHa and Caski cells, of which the inhibition of E7 expression resulted in the up-regulation of tumor suppressor protein pRb, eventually inducing tumor cell apoptosis and inhibiting tumor cell growth [58]. In addition, targeting the promoter of HPV-16 E6/E7 using the similar approach in SiHa cells also inhibited the mRNA and protein expression levels of E6/E7 and up-regulated the expression of tumor suppressors p53 and p21, which inhibited the in vitro proliferation of SiHa cells and the in vivo growth of a subcutaneously transplanted tumor in a NOD/SCID tumor mice model [59]. Recently, a study using the CRISPR/Cas9-mediated loss of E7 from HPV-associated oropharyngeal squamous cell carcinomas (OPSCC) cells, SCC2 and SCC104, was shown to restore cGAS-STING responses, the activation of which may induce favorable tumor clearance [60]. These studies have suggested that the CRISPR/Cas9 system may be an effective strategy for treating HPV-related tumors.

As a human mouse mammary tumor virus like-2 (HML-2) subgroup of human endogenous retroviruses (HERVs), the HERV-K is activated in several tumors and has been suggested to be related to prostate cancer progression and motor neuron diseases. In a recent study [61], the HERV-K env gene, a retroviral gene with oncogenic and neuropathogenic potential, was disrupted by the CRISPR/Cas9 technology and was demonstrated to interfere with important regulators of gene expression and proliferation of human prostate cancer LNCaP cells. It implies that HERV-K is not an innocent bystander and reinforces its link to oncogenesis and motor neuron diseases, opening a potential innovative option for future therapy.

#### 4.1.3. Herpesvirus Gene Editing Using CRISPR/Cas9 System

Herpesviruses are double-stranded DNA viruses with large genomes. In the infected host, herpesviruses have the distinct ability to escape the surveillance of the host immune system by establishing a life-long latent infection and causing recurrent diseases. Herpesviruses include a variety of important human pathogens, such as herpes simplex virus (HSV), Epstein–Barr virus (EBV), human cytomegalovirus (HCMV), and livestock and poultry pathogens such as pseudorabies virus (PRV), duck enteritis virus (DEV), infectious laryngotracheitis virus (ILTV), and Marek’s disease virus (MDV).

The CRISPR/Cas9 system can achieve direct DSB in the genome of DNA viruses, using NHEJ and/or HDR pathways to introduce site-specific indels or insertion of heterologous genes with high frequency [62,63]. In 2014, the CRISPR/Cas9 system was reported for insertion of foreign genes into an adenoviral vector and type I HSV (HSV-1) with only one round of selection, changing the genomes of large DNA viruses and interfering with virus replication [64]. The genome mutation efficiency of recombinant progeny virus reached 47–52%, and the homologous recombination efficiency rate increased to 2.6 ± 0.57%. This is the first report that showed that the CRISPR/Cas9 system could be applied to edit the genome of large DNA virus, making the construction and purification of recombinant progeny viruses easier.

The CRISPR/Cas9 system has recently been reported as an antiviral strategy to interfere with human herpesvirus infection in vitro [65]. The gE and TK genes have been successfully deleted from the genome of HSV, and the reverse mutation in gE-deleted strains has also been realized by the CRISPR/Cas9 system [66]. Subsequently, it was reported that the in vitro replication of EBV, HCMV, and HSV-1 could be significantly inhibited by transferring specific gRNA into the cell model by the CRISPR/Cas9 system [67]. Some scholars have used the CRISPR/Cas9 system to target the ICP0, ICP4, and ICP27 genes to completely inhibit the replication of HSV-1 in host cells [68]. The targeted mutation of another viral protein, UL7, a tegument protein of HSV-1, can attenuate the neuro-virulence of the virus by reducing the modulation of *α*-4 gene transcription [69]. In addition, the combination of the CRISPR/Cas9 technique and flow cytometry has increased the HDR efficiency of recombinant HSV DNA by 10,000–1,000,000 times [70]. These results can have an important impact on the study and gene therapy of HSV.

Some researchers have explored the role of the CRISPR/Cas9 system in EBV gene editing. Seven gRNAs were designed and transfected into Raji cells with latent EBV infection. It has been demonstrated that once the corresponding functional sites of the EBV viral gene were destroyed, the cell proliferation and virus load were both significantly decreased, whereas the apoptosis pathway in the cells was restored [71]. Deletion of a 558 bp fragment in the BART promoter region of EBV was realized using two gRNAs mediated by the CRISPR/Cas9 system [72]. The gRNAs targeting different regions of the EBV genome were designed and transfected into C666-1 cells, which resulted in the decrease of EBV DNA by about 50% and further confirmed the feasibility of the CRISPR/Cas9 system in EBV gene editing. Although the suppression of EBV did not affect the survival of c666-1 cells, the cells were sensitive to cisplatin and 5-fluorouracil, suggesting that the CRISPR/Cas9 system may be a potential strategy to make EBV-transformed cancer cells more sensitive to chemotherapy drugs [73].

Many scholars have applied the CRISPR/Cas9 system to the gene editing of other human herpesviruses too. Three specific gRNAs targeting the UL122/123 gene of HCMV, a key regulator responsible for lytic replication and reactivation from latency, were transfected into primary fibroblasts and U-251 MG cells [74]. A concomitant reduction of immediate-early (IE) protein expression was induced, and the late protein expression and virus replication were reduced by 90%. Finally, the replication of new HCMV virus particles was significantly prevented. The CRISPR/Cas9/sgRNA lentiviral constructs were recently used to target the IE region of the HCMV genome, which significantly reduced the viral gene expression and virion production in HFF primary fibroblasts and inhibited the viral DNA production and reactivation in the THP-1 monocytic cell line [75]. A replication-incompetent adenovirus type 5 (Adv5), delivering a latency-associated nuclear antigen (LANA)-specific Cas9 system (Ad-CC9-LANA) to target the LANA of Kaposi’s sarcoma-associated herpesvirus (KSHV) gene products, was designed to transfect various KSHV latent cells and has disrupted the latency in KSHV-infected epithelial and endothelial cell lines [76]. This approach to limit the latency of KSHV may also represent a viable strategy against other tumorigenic viruses. Thus, the CRISPR/Cas9 system can effectively target a variety of herpesvirus genomes that cause human diseases, significantly inhibiting virus replication and providing a new idea for the treatment of diseases.

In addition to human disease-related herpesvirus, the CRISPR/Cas9 system is also attractive for scientists focusing on animal herpesvirus. In 2015, some researchers co-transfected the purified PRV genomes with the constructed specific gRNA CRISPR/Cas9 complex into PK15 cells and obtained up to 100% viral gene editing efficiency [77]. Simultaneously, a cell line stably expressing Cas9 nuclease and sgRNA targeting the UL30 gene conserved in PRV was developed, finding that the UL30 gene of infected PRV can be cleaved efficiently, and the stable expression of Cas9 nuclease has no adverse effect on the proliferation of PRV [78]. Compared to the single gRNA-based transfection-infection approach, the double-gRNA strategy demonstrated a significantly better knockout or knockin efficiency for manipulating PRV viral genes [79]. Furthermore, a total of 75 sgRNAs targeting both of the essential and non-essential genes across the genome of PRV were designed and transfected into Vero cells, and most of them showed significant inhibition of PRV infection and replication [80]. Using the double-gRNA strategy, the meq and pp38 genes of serotype 1 MDV (MDV-1) were also successfully knocked out from the viral genome of CVI988/Rispens vaccine strain, showing no obvious influence on virus replication [81]. More recently, the CRISPR/Cas9 approach was used as a screening tool for identifying essential viral genes that could be used to block MDV replication as a future tool for protecting chickens against MDV infection [82]. CRISPR/Cas9-based editing has also been extended into the integrated viral genomes of MDV-transformed lymphoblastoid cell lines. Deletion of the pp38 gene from the MDV genomic DNA integrated into the host genomes of virally transformed T-lymphoma cell lines (MDCC-MSB-1 and MDCC-HP8 cells) by the CRISPR/Cas9 system showed an increase in the proliferation of tumor cells, indicating that pp38 is not necessary for the transformation of T-lymphoma cell lines [83]. Furthermore, a series of virus-encoded microRNAs (miRNAs) in the viral genomes of MDV-1 strain RB-1B or T-lymphoma MSB-1 cell line was successfully mutated by CRISPR/Cas9 system [84,85], providing new important clues for revealing the regulatory roles of viral tiny RNAs in triggering the virally induced T cell lymphomagenesis.

#### 4.1.4. Other DNA Virus Gene Editing Using CRISPR/Cas9 System

Recently, the CRISPR/Cas9-based gene-editing system has also been successfully applied in other DNA viruses, such as the vaccinia virus (VACV), JC polyomavirus (JCPyV), fowl adenovirus 4 (FAdV-4), and African swine fever virus (ASFV). The gRNA-guided CRISPR/Cas9 system has greatly increased the efficiency of generating mutant VACVs without evident off-target effects, providing a marker-free system that can be used for the efficient construction of VACV vectors armed with any therapeutic genes in the TK or N1L regions [86,87,88,89]. The system targeting the non-coding region and late gene open reading frame (ORF) in the JCPyV genome inhibited the virus replication and viral protein expression [90]. In a permissive wild boar lung (WSL) cell line, the viral plaque formation of ASFV was completely abrogated, and virus yields were significantly reduced by the stable expressed Cas9 and a guide RNA targeting codons 71 to 78 of the phosphoprotein p30 gene (cp204l) [91]. With the CRISPR/Cas9 system, a unique 1966-bp nucleotide-deletion (1966DEL) of a non-pathogenic FAdV-4 strain KR5 was inserted into the loci between ORF42 and ORF43 of the highly pathogenic FAdV-4 strain (HLJFAd15) with a natural deletion of 1966DEL to rescue the recombinant Re1966 strain, the results of pathogenicity study confirmed that the natural 1966DEL was not an essential site for the increased virulence of FAdV-4 [92].

#### 4.1.5. RNA Virus Gene Editing Using CRISPR/Cas9 System

At present, the CRISPR/Cas9 system is mainly used for DNA virus gene editing, and there are still huge technical obstacles in its application in RNA virus mutagenesis. A new Cas9 nuclease from the Gram-negative bacterium *Francisella novicida* (FnCas9) has demonstrated the ability to target endogenous bacterial RNA, and the reconstructed gRNA targeting human +ssRNA virus (such as HCV) in eukaryotic cells inhibits the expression of viral proteins, providing a possibility for the treatment of diseases caused by RNA virus infection [93]. The latest developed CRISPR/Cas13a system was recently reported to be capable of efficiently targeting the NS3 region of dengue virus (DENV) and inactivated virus replication in mammalian cells [94]. Infectivity abrogation of porcine reproductive and respiratory syndrome virus (PRRSV) was also successfully developed by targeting viral ORF5 and ORF7 genes using the CRISPR/Cas13b-system, and the viral RNA cleavage was observed in mammalian cells [95]. These data demonstrate a novel and effective technology for future gene editing of RNA virus.

#### 4.1.6. Virus–Host Interaction Studies Using CRISPR/Cas9-Based Gene Editing

Recently, genome-wide CRISPR/Cas9 screening has been used to identify host factors that are potentially involved in the replications of some viruses, such as the severe acute respiratory syndrome coronavirus 2 (SARS-CoV-2) [96], HIV [97], arthritogenic alphaviruses [98], Coxsackievirus [99], Venezuelan equine encephalitis virus (VEEV) [100], and influenza virus [101]. For the influenza virus, it has been demonstrated that inactivation of the specific host genes, such as DOCK5, Annexin-A1, IFIT2, IRF7, and ZDHHC22, can induce protection against cell death by influenza virus infection [102,103,104,105,106]. With the advantages of having a high throughput and allowing the precise editing of the CRISPR/Cas9 screening system, we can quickly and accurately identify the specific genes and proteins contributing to the pathogenesis of various viruses. The relative ease of use and reproducibility of the CRISPR/Cas9 system make it a powerful tool for probing virus–host interactions and for identifying new antiviral targets in future virology research.

### 4.2. Application of CRISPR/Cas9-Based Gene-Editing Technology in Vaccine Development

In addition to the studies on gene function, gene therapy, and virus–host interaction, CRISPR/Cas9-based gene-editing technology has also been widely applied in vaccine research for its high efficiency, specificity, versatility, flexibility, simplicity, and low cost compared to the other viral genome editing techniques, which has demonstrated an efficient and powerful strategy for future development of genetically engineered vaccines. The latest progress in the application of CRISPR/Cas9 in virus genome editing and advancing orthopoxvirus (OPXV)-based recombinant vaccines and vectors has been well summarized by Okoli and colleagues [107]. A CRISPR/Cas9 genome-wide screening strategy to identify and rank host restriction factors of H1N1 influenza virus in a cell-based vaccine production platform has raised the prospect for future increases in vaccine yield [101].

Recently, the number of studies on animal anti-viral vaccine development utilizing the CRISPR/Cas9 system has been increasing (Table 1). The CRISPR/Cas9 system-mediated knockin of more than 4-kb-long DNA cassettes into the PRV genome at a positive rate of 50% by NHEJ, making PRV a promising vector for the development of vaccines [77]. In a later study, a vaccine candidate strain with double-deletion of TK and gE genes of PRV was successfully constructed by the CRISPR/Cas9 system [108]. Similarly, a triple gE/gI/TK gene-inactivated HeN1 PRV strain has been demonstrated to be fully attenuated and can provide immune protection against parental PRV challenge [109]. Based on a PRV variant (NY strain), a triple gE/gI/TK-deleted mutant has been recently constructed through homologous DNA recombination and CRISPR/Cas9-based gene-editing technology, providing a next-generation vaccine candidate for control of potential new variant PRV strains [110]. Using the CRISPR/Cas9 system, knockout of non-essential gene 8-DR from the genome of ASFV virulent strain Georgia07 in porcine macrophages was also reported to successfully rescue the recombinant virus, which laid a foundation for the follow-up vaccine development for future control of African swine fever [111].

In 2016, CRISPR/Cas9-mediated genome editing in the live attenuated vaccine vector herpesvirus of turkeys (HVT) was reported as the first application of this technology in avian herpesvirus mutagenesis [112]. Based on the CRISPR/Cas9 and NHEJ system, a red fluorescent protein (RFP) expression cassette was inserted into the UL45/UL46 region of the HVT genome to construct a general donor plasmid, and then the inserted RFP gene was removed using the Cre-Lox system and the VP2 gene of infectious bursal disease virus IBDV was inserted into the UL45/UL46 region to construct a recombinant HVT/IBDV-VP2 vaccine [113]. Then, ILTV gD/gI and avian influenza virus (AIV)-H9N2 hemagglutinin (HA) gene expression cassettes were inserted into the 065/066 and US2 regions of HVT/IBDV-VP2 recombinant virus, resulting in a triple inserted HVT-VP2-gDgI-HA vaccine candidate [114]. Some scholars have tried to use the CRISPR/Cas9 and HDR systems to insert the AIV H7N9-HA expression cassette into the same UL45/UL46 site in the HVT genome, successfully constructing an HVT-H7N9-HA candidate bivalent vaccine [115]. In addition, the other researchers have inserted the H9N2-HA gene of the AIV BJ/15 strain into the UL45/UL46 region of the HVT-BAC genome and deleted the residual fragments of HVT-BAC using the CRISPR/Cas9 system to generate a recombinant rHVT-H9N2-HA vaccine strain [116].

Simultaneously, some scholars have applied this technology to perform similar research on other poultry herpesviruses. The CRISPR/Cas9 and HDR systems were used for the insertions of the HA gene of highly pathogenic AIV H5N1, the pre-membrane (PrM), and gE genes of duck Tembusu virus (DTMUV) into the UL27/UL26 and US7/US8 regions of the DEV C-KCE strain genome, to develop a potential trivalent vaccine to prevent the infections of H5N1-AIV, DTMUV, and DEV [117]. Subsequently, AIV H5N1-HA was inserted into the UL27/UL26 region of the DEV genome using the NHEJ-CRISPR/Cas9 and Cre-Lox systems to produce a recombinant DEV-AIV vaccine [118]. Using the same strategy, the thymidine kinase (TK) and unique short 4 (US4) genes were deleted from the viral genome of infections laryngotracheitis virus (ILTV), and the insertion of fusion (F) gene of Newcastle disease virus (NDV) demonstrated no adverse effects on ILTV replication and expression of the F protein [119]. This is the first attempt to knock out virulence factors and insert heterologous genes into the viral genome to generate a multivalent recombinant vaccine. These studies confirmed that the CRISPR/Cas9 system can be used for the rapid and efficient genome modification and recombinant vaccine development of avian herpesviruses.

CRISPR/Cas9-based genome editing has also been applied in generating a vaccine against canine infectious disease. For both wild and domestic carnivores, canine distemper (CD) is one of the most important infectious diseases caused by the canine distemper virus (CDV). Using the CRISPR/Cas9-based gene editing approach, a highly efficient recombinant virus, containing CDV virus-like particles (VLPs) and concurrently expressing the matrix (M), H, and F genes, has been recently constructed, which can assemble CDV-VLPs and provide faster seroconversion and higher rates of antibody positivity than the parental virus strain among foxes and minks [120]. This is the first report suggesting that the CRISPR/Cas9 system can be applied for rapid and efficient vaccine development for the prevention of CD among dogs, foxes, and minks in the future.

## 5. Prospects

Recent advances in the development of CRISPR/Cas9-based gene-editing technology has brought immense opportunities for molecular interrogation of viral genetic determinants of virus–host interactions, pathogenicity, gene-deleted, and/or recombinant vaccines, through its special advantages of simple design, high efficiency, low cost, and wide application; however, some shortcomings, especially the possible occurrence of “off-target” effects, are causing some concerns. According to the principle of complementary base pairing, gRNA guides Cas9 nuclease to carry out strict site-specific cleavage of target genes; however, within the whole genome, the binding of gRNA to non-specific target sites may lead to an unexpected genomic modification, which is called “off-target”. Therefore, the possibility of “off-target” occurrences should be first considered to be avoided or reduced as much as possible when gRNAs are analyzed and designed. To resolve this problem, scientists have established various strategies to increase the gRNA specificity to eliminate the occurrence of “off-target” modification, such as using recombinant Cas9 nuclease and/or Cas endonuclease from different bacterial sources, shortening the length of the gRNA sequence, or making some changes in the secondary structure. All these measures have been able to reduce “off-target” modifications to a certain extent. We can also analyze, monitor, and prevent the occurrence of “off-target” modifications with the help of computational software prediction, intra- and extracellular detection, and whole-genome sequencing.

Until now, most of the reports on the CRISPR/Cas9-based gene editing of viruses are still in the stage of laboratory research and are focusing on three main aspects: (1) using this technology to achieve the deletion of viral pathogenic genes as potential tools for gene therapy applications against diseases, although this still requires long-term research, observation, and practice to realize the clinical application; (2) editing some of the functional viral genes, as a simple and rapid genetic engineering technology to be applied for studying the virus pathogenesis and/or virally induced tumorigenesis; (3) modifying the viral genome by gene deletion, substitution, and/or insertion to construct vaccine candidate strains with various gene recombination, providing a new efficient approach for the research and development of recombinant vaccines. However, a long period is required to observe the precise editing site and the passage stability of reconstituted viruses. The emergence and development of any innovative technique is a key step in progressing both science and technology. Undoubtedly, CRISPR/Cas9-based gene editing technology provides endless possibilities for future scientific research and vaccine development to meet the significant outstanding challenges in biology.

## Figures and Tables

**Table 1:** Virus species reconstituted for animal vaccine development by the CRISPR/Cas9-based gene editing techniques.

Viruses *	Serotype/Strain	Target	Classification	Proposed Usage	Reference
PRV	BarthaK61	EP0, UL50	Insertion	Knockin of >4-kb-long DNA cassettes into the PRV genome for expressing foreign genes.	[77]
	HNX	TK, gE	Deletion	Double-gene deletion for vaccine development.	[108]
	HeN1	TK, gI, gE	Mutation	Triple-gene deletion for live attenuated vaccine development.	[109]
	NY	TK, gI, gE	Deletion	Triple-gene deletion for live attenuated vaccine development.	[110]
ASFV	Georgia07	8-DR	Deletion	Attenuated ASFV vaccine development.	[111]
HVT	FC-126	gB, gI, gE	Mutation	Development of new, highly immunogenic, multivalent vectored vaccine.	[112]
		UL45/46	Insertion	Development of recombinant vaccine candidates HVT-IBDV-VP2.	[113]
		UL45/46, HVT65/66, US2	Insertion	Generation of a triple insert HVT-VP2-gDgI-HA recombinant vaccine against MDV, ILTV and AIV.	[114]
		UL45/46	Insertion	Constructs a bivalent HVT-H7N9-HA candidate vaccine strain.	[115]
		Residual fragments of HVT-BAC	Deletion	Constructs a recombinant rHVT-H9N2-HA candidate vaccine strain.	[116]
DEV	C-KCE	UL27/UL26, US7/US8	Insertion	Constructs a potential trivalent vaccine strain C-KCE-HA/PrM-E to prevent H5N1-AIV, DTMUV and DEV infections.	[117]
	VR-684	UL27/UL26	Insertion	Constructs a recombinant bivalent DEV-AIV vaccine.	[118]
ILTV	GaHV-1 strain	UL22/UL27, UL47/UL4	Deletion/Insertion	Deletion of the TK & US4 genes; insertion of the NDV-F into ILTV for constructing a stable vaccine vector.	[119]
CDV	ALVAC, a CNPV vector	H, F, M genes	Insertion	Construct a recombinant virus ALVAC-CDV-M-F-H/C5−assembling CDV-VLPs and concurrently expresses the matrix (M), H, and F genes.	[120]

* PRV: pseudorabies virus; ASFV: African swine fever virus; HVT: herpesvirus of Turkeys; MDV: Marek’s disease virus; DEV: duck enteritis virus; ILTV: infections laryngotracheitis virus; CDV: canine distemper virus; GaHV-1: Gallid alphaherpesvirus 1.
